# A case report of inhalation anthrax acquired naturally

**DOI:** 10.1186/s13104-016-1955-0

**Published:** 2016-03-03

**Authors:** Zohreh Azarkar, Majid Zare Bidaki

**Affiliations:** Medical Faculty, Hepatitis Research Center, Birjand University of Medical Sciences, Brijand, Iran; Hepatitis Research Center, Medical Microbiology Department, Paramedical Faculty, Birjand University of Medical Sciences, Ghafari Avenue, Birjand, South Khorasan Iran

**Keywords:** *Bacillus anthracis*, Inhalation anthrax, Natural acquisition, Case report

## Abstract

**Background:**

Anthrax is a zoonotic occupational disease caused by *Bacillus anthracis*, a rod-shaped immobile aerobic gram-positive bacteria with spore. Anthrax occurs in humans randomly and with low frequency. Most cases of anthrax are acquired through contact with infected animals or contaminated animal products. This old disease became particularly important since 2001 that the biological spores were exploited in America. Depending on the transmission method of the disease, clinical manifestations occur in three classes: Cutaneous, respiratory, and gastrointestinal anthrax. The respiratory form is considered as the most fatal and a rare form of anthrax intending to show complicated and unusual manifestations.

**Case presentation:**

In this case report a rare case of inhalation anthrax acquired naturally in southeast of Iran is presented. A blind 65-year-old man, living in a rural area, was admitted with respiratory infection, fever, dyspnea, loss of appetite, and myalgia. The patient was treated with outpatient antibiotics a week ago. After admission, the patient was again treated for pneumonia, but there was no improvement despite treatment and the patient was suffering from septicemia symptoms. Radiographic images showed wide mediastinum. *Bacillus anthracis* was isolated from blood and sputum culture and the results were confirmed by colony morphology, biochemical reactions and PCR. The treatment was changed to ciprofloxacin, clindamycin, and penicillin. On the second day of anthrax treatment, the patient was complicated with jaundice, elevation of liver enzymes, and a significant drop in hemoglobin, hematocrit, and platelet despite lack of obvious bleeding and was complicated with respiratory distress and sepsis and died a week after treatment.

**Conclusions:**

We could discover no specific exposure associated with anthrax infection for this patient. However, due to being located in an endemic and enzootic area, it is proposed that the exposure occurred through contact with infected airborne dust or an unknown contaminated item. Despite many advances in preventing anthrax, still some rare cases of respiratory and complicated anthrax are emerging. With regard to the threat of bioterrorism, medical staff’s sensitivity to the clinical syndrome, methods of prophylaxis and treatment of anthrax must be raised. Fast diagnosis and successful treatment the lethal cases of this infection are of utmost important.

## Background

Anthrax is a zoonotic occupational disease caused by *Bacillus anthracis*, a rod-shaped immobile aerobic gram-positive spore former bacterium. The spores can survive in the soil for years and primarily infect vegetarians. Anthrax occurs in humans randomly and with low frequency. No known cases of direct transmission of its causative agent, *B. anthracis*, from human to human has been reported, but the infection is acquired through contact with infected animals or contaminated animal products [1, 2]. Due to the frequency of infection in workers exposed to contaminated animal products, in nineteenth century respiratory anthrax cases were called woolsorter’s disease. [1].

This old disease became particularly important since 2001 that the biological spores were exploited in America. Depending on the transmission method of the disease, clinical manifestations occur in three classes: cutaneous, respiratory, and gastrointestinal anthrax, 95 % of which include the cutaneous form [1–3] and the respiratory form is considered as the most fatal form of anthrax reported which can lead to fatal septicemia. However, some other unusual manifestations have also been reported [2, 4]. In this report a rare case of inhalation anthrax acquired naturally is presented. This may underline the challenges of working on anthrax cases when no exact source of the infection exists.

## Case presentation

The patient was a 65-year-old blind man, living in countryside of Birjand, the center of South Khorasan province located in southeast of Iran. The case-patient mainly lived alone in the past 3 months due to his wife’s death. He had a history of keeping animals including some sheep and goats at home, but his animals were not infected. He had a long history of COPD. But his symptoms had started a week ago. The symptoms had not improved despite antibiotic treatment and therefore, the patient was referred to us at the medical center of the province. He characterized with mild fever, dyspnea, pussy non-bloody sputum, anorexia, and myalgia. In primary examination, he had a blood pressure of 120/80 mmHg, respiratory rate of 24 per minute, pulse rate of 90 beats per minute, and a temperature of 37.6 °C. In physical examination, he had no skin rash, ulcer, and necrosis in his face and extremities, and also no symptoms of icterus were observed. Lung auscultation revealed a slightly hoarse voice. The patient had no signs of meningeal irritation and chest x-ray was normal.

Initial tests of the patient were as follows: White blood cell = 7.3 × 10^3^/µL, Neutrophils = 85 %, Hemoglobin = 13.2 g/dl, Hematocrit = 42.2 %, Platelet = 137 × 103/µL, ESR = 19, CRP = 2+, Urine analysis = normal.

By physical examination, patient was diagnosed as exacerbation of COPD or pneumonia and received intravenous ceftriaxone and azithromycin. Despite 3 days of treatment, his symptoms did not change and the patient became slightly disoriented. Because of the intermittent sleepiness, lack of signs of meningeal irritation and lack of his next of kin consent, the patient did not undergo lumbar puncture, but blood samples were sent to the laboratory for examination.

The first blood sampling was taken on the 1st day of admission but the results were reported to the ward on the 3rd day. According to laboratory reports, microscopic blood samples revealed presence of gram-positive streptobacilli. Blood culture on blood agar showed growth of creamy coarse grayish texture with irregular borders colonies without hemolysis looking like a jellyfish. In the next stage, by conducting a number of microbial enzymatic and metabolic tests, including motility, gelatin hydrolysis, bacterial sensitivity to penicillin, string of pearls test, sugars fermentation, *B. anthracis* was strongly suggested. All these routine laboratory procedures took 48 h.

On the 3rd day of hospitalization, the chest x-ray of the patient was repeated, which showed closed right side angles, wide mediastinum, and opacities in lower region of the lung.

With regard to blood culture results and x-rays of the patient, the antibiotic was changed to ciprofloxacin, clindamycin, and penicillin. Moreover, the patient’s blood and lung secretion samples were sent to the National Reference Laboratory to repeat the culture and subsequently to do PCR.

On the 2nd day of anthrax treatment, the patient was complicated with jaundice, elevation of liver enzymes, and a significant drop in hemoglobin, hematocrit, and platelet despite lack of obvious bleeding and this was complicated with respiratory distress and sepsis. The patient was finally died a week after treatment. Autopsy was not performed because of discontentment of patient’s family. The organism isolated repeatedly from the blood and from the lung secretion, and the results of PCR confirmed respiratory anthrax and the subsequent sepsis.

Reporting this critical finding to public health department, a group of health care workers were assigned to mission visiting the case-patient’s rural house and carrying out a disinfect process there. During the visit, they found an unprocessed sheepskin used as flooring. However, bacteriological analysis of samples taken from the sheepskin did not reveal any trace of *B. anthracis*. Environmental sampling was not conducted for *B. anthracis*.

## Discussion

Anthrax is a zoonotic disease. Epidemiologic studies and case reports have shown that most cases of anthrax infection occurs in people living in rural areas or in occupations related to animal products [5–7]. The most common form of anthrax infection in humans is the cutaneous form that is diagnosed by a topical skin lesion with central scar and marked non-pitting edema.

In contrast, the prevalence of respiratory anthrax infection is very low; it usually begins with acute signs and respiratory symptoms such as dyspnea, hypoxia, and hypotension occurs 1–3 days after the onset of the disease that results in death within a short time. Sometimes patients are discovered with bleeding mediastinitis in autopsies [1, 2]. In cases where these organisms enter the bloodstream and septicemia occurs, the number of bacteria per cubic centimeter of blood can ultimately reach more than 10^8^ before the patient’s death.

In the early 1900s, the human cases of respiratory anthrax in the United States occurred mainly in those who had contact with textiles and leather industry and was named as Wool sorter disease. In the late twentieth century, with the increase in health level and protection of animal products, the disease prevalence was reduced, but the death cases were still very high (>85 %). In 1979, in the Sverdlovsk event in an industrial zone in the former Soviet Union, an epidemic of respiratory anthrax occurred due to the recklessness in the military industry. In 2001, ten cases of deliberate infection of respiratory anthrax were confirmed that was due to a bioterrorist attack on the United States, in which individuals were targeted with packets of *B. anthracis* spores [8, 10]. Whereas, only 18 cases of respiratory anthrax was reported in the US prior to that, the latest of which happened in 1976 [8, 9]. Moreover, a systematic review conducted in 2006 showed that the total number of inhalation anthrax cases reported in any languages between years 1900 and 2005 was 82 [10]. This apparently reveals the rare incident of this disease in general. Therefore, reports of anthrax is currently of particular importance in the whole world. Most cases of anthrax reported in Iran include cutaneous, gastrointestinal and meningitis types [5–7]. Based on the results of medical databases, this was the first respiratory anthrax over the last 30 years in Iran.

Respiratory anthrax starts with feeling unwell, myalgia, fatigue, fever, nonproductive cough and then more severe respiratory distress and cyanosis occurs and the patient usually dies within 1–2 days. In this case report, the onset of symptoms was respiratory and gradually progressed. The patient was complicated with jaundice, elevated liver enzymes, and respiratory distress after 5 days. Regarding the mediastinal edema in patients suffering from respiratory anthrax, which is due to *bacillus anthrax* secreting edema factor, chest x-ray is considered a sensitive measurement in the diagnosis of respiratory anthrax. Respiratory manifestations occur in several forms in patients, including mediastinal widening, involvements around trachea, hilar infiltration, pleurisies, and parenchymal infiltration [1, 11].

In this case, the initial chest x-ray was normal, but after a few days infiltration, pulmonary edema, pleurisies, and mediastinal widening was observed, despite respiratory symptoms did not deteriorate. Number of white blood cells is normal or slightly increased in respiratory anthrax. Particularly, increased neutrophils can help the diagnosis of the disease primarily [11]. However, no increase in the number of leukocytes, especially neutrophils were observed in the reported patient.

In animals with respiratory anthrax infection, bacteremia is reported in the early phase of the disease before fulminant symptoms. But blood culture rapidly becomes negative after antibiotic treatment. Administration of antibiotics before blood culture results in negative blood culture, which is the most definitive method of diagnosis [9]. Nevertheless, blood and sputum cultures of the reported case were still positive after 3 days of antibiotic therapy.

Natural acquisition of anthrax’s means transmission of disease through contact with infected animals or animal products to human. The last known case of anthrax respiratory disease acquired normally was in America in 1976 [12]. Thereafter, the involvement of respiratory anthrax has been usually due to bioterrorism or after sepsis caused by cutaneous wounds. But in our case, natural acquisition of respiratory anthrax occurred without injury or risk of bioterrorist attack. The source of the disease was not detected in the medical history of the patient, but it seemed that the patient acquired anthrax due to contact with animals and their products. “As a villager blind, he used to touch and smell anything for finding his wanted items around”, Said our patient’s son to health care workers. The above sentence may show a strong clue proposing the way that the case-patient was infected. It seems that he acquired anthrax due to adjacent contact with animals’ products, especially with wool. Soil might be the other alternative source of this contamination. A previous research conducted on determination of anthrax foci throughout Iran, southeast of the country (Khorasan region) was demonstrated as one of the endemic regions [13]. Intensive rain or drought leads the spores to bud and reproduce *B. anthracis* that cause the bacteria to multiply enough to cause infection [1, 9]. Considering the prolonged droughts in the border areas of East Iran, drought is perhaps one of the reasons of occurrence of this disease.

The survival of patients with respiratory anthrax is reported 60 % in some studies and higher in some other. This patient was treated with a combination of antibiotics, effective against *B. anthracis* (Ciprofloxacin 400 mg q12 h IV, Clindamycin 600 mg q8 h IV, and Penicillin 2 MU q2 h IV).

Resistance to Penicillin has increased and the drug is not used in this occasion in many parts of the world. However, antimicrobial susceptibility testing showed that the isolate was totally sensitive to Penicillin. Moreover, Penicillin is the drug of choice to treat coutanous anthrax in this region and we have had previous successful experiences.

*BacillusAnthracis* generates cephalosporinase that can inhibit the activity of cephalosporins, such as ceftriaxone. Therefore, cephalosporin should not be used in the treatment of this disease. Early detection and treatment, proper antibiotics, differences in the pathogenesis of anthrax cases caused by bioterrorism, host sensitivity or a combination of these factors contribute to patient survival [1, 9]. Administration of anti-anthrax antiserum has shown to improve some human cases of anthrax. In anthrax generated in animal models, the best treatment for anthrax infections is appropriate antibiotic and antiserum. Also, in some cases, a combination of human immunoglobulin and antibiotic administration were effective. [10, 12].

Further studies are needed to detected adjuvant therapies, such as human immunoglobulin and anti-toxin, corticosteroids, and other toxin inhibitors in the treatment of anthrax.

Respiratory anthrax should be considered in differential diagnosis of patients with severe pulmonary involvement and in endemic areas due to its mortality and similarity of its symptoms to lower and upper respiratory infections and other respiratory involvements.

## Comments and conclusions

We could discover no specific exposure associated with anthrax infection for this patient. However, it is proposed that the exposure occurred through contact with infected airborne dust or an unknown contaminated item. Immunodeficiency and chronic pulmonary disease can increase the susceptibility to inhalation anthrax [14]. This old patient had a long history of COPD, possibly this increased his risk.

We advise that inhalation anthrax should be considered in patients with severe pulmonary involvement, especially in endemic regions due to its high rate of mortality and also the similarity of its symptoms to lower and upper respiratory infections and other respiratory involvements.

In spite of enhancement in preventing anthrax, still some rare cases of respiratory and complicated anthrax are emerging. These cases are found much more in endemic end enzootic regions of the world. With regard to the threat of bioterrorism, medical staff’s sensitivity to the clinical syndrome, methods of prophylaxis and treatment of anthrax must be raised. Fast diagnosis and successful treatment of inhalation and sepsis cases of anthrax are of utmost important.

This case study was limited by lack of soil and environmental sampling. The other limitation was lack of determination of MIC for the prescribed antibiotics.

## Abbreviations

Hb: hemoglobin
HCT: hematocrit
Plt: platelets
ESR: erythrocyte sedimentation rate
CRP: C-reactive protein
U/A: urine analysis
COPD: chronic obstructive pulmonary disease
